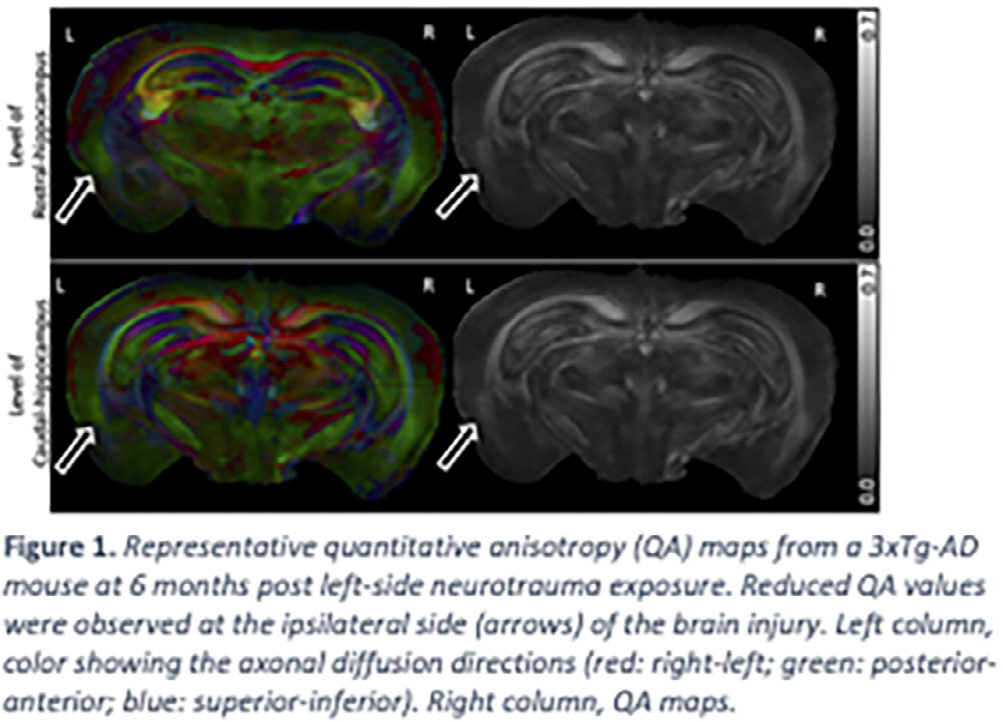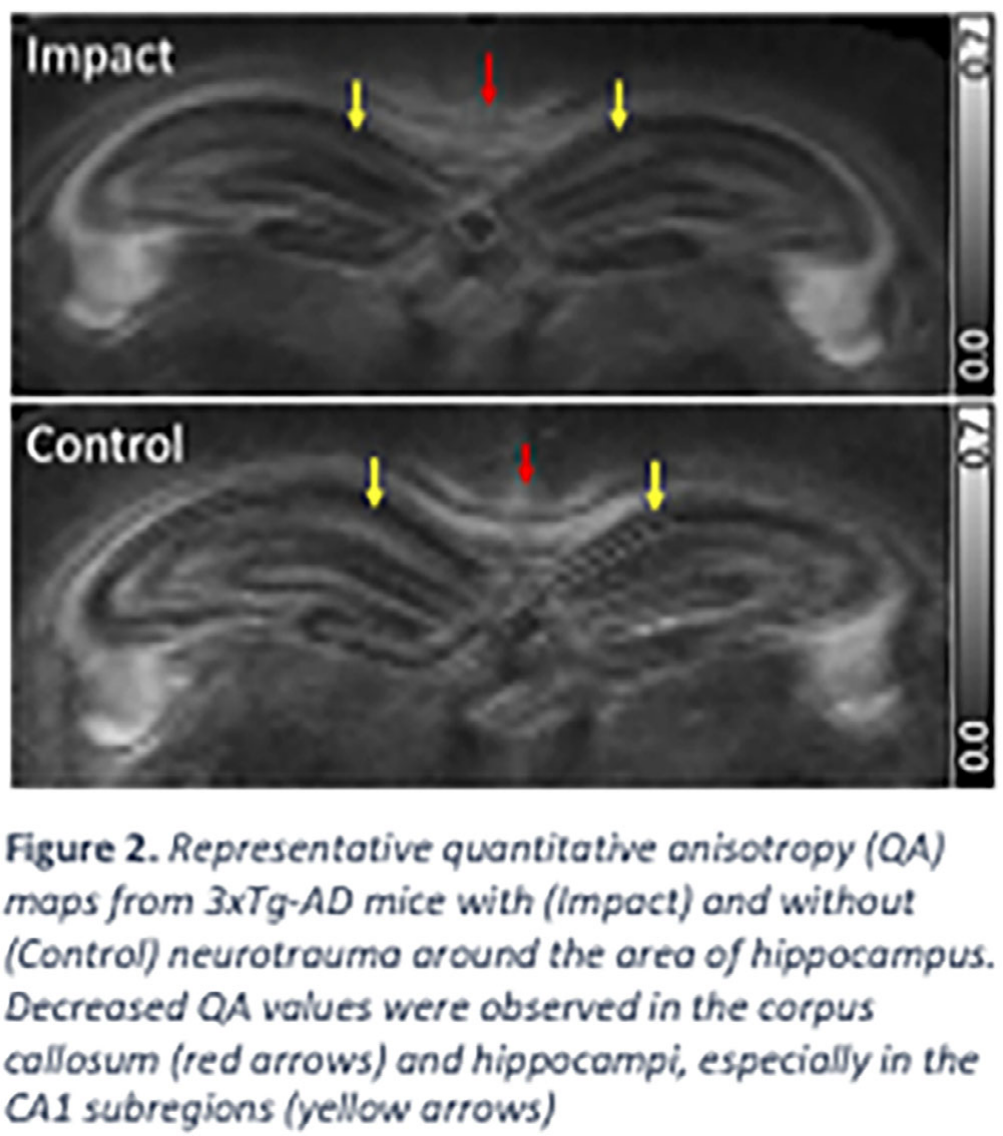# Application of High‐Resolution Diffusion‐MRI in Neurotrauma Accelerated Alzheimer's Disease

**DOI:** 10.1002/alz.089758

**Published:** 2025-01-09

**Authors:** Ning Hua, Olga Minaeva, Douglas Parsons, Juliet A Moncaster, Hernan Jara, Lee E Goldstein

**Affiliations:** ^1^ Boston University Chobanian & Avedisian School of Medicine, Boston, MA USA; ^2^ Boston University Alzheimer's Disease Research Center, Boston, MA USA; ^3^ Boston University Alzheimer’s Disease Research Center, Boston, MA USA

## Abstract

**Background:**

Traumatic brain injury (TBI) is a risk factor for earlier onset of Alzheimer’s disease (AD)( Graves AB, et al., 1990), and the more severe the injury, the greater the risk of developing AD(Johnson VE, et al.,2010). Given the prevalence of AD in modern society, the possibility that TBI may predispose individuals to develop AD has significant social and economic implications. Therefore, it is important to understand how TBI triggers accelerated AD progression. In this study, we explored how neurotrauma accelerated white matter degeneration in a transgenic mouse model of AD using high‐resolution (60mm^3^) ex vivo diffusion MRI.

**Method:**

3xTg‐AD mice were pretreated with a non‐sedating dose of the analgesic buprenorphine (0.2mg/kg, i.p.) prior to TBI. 3xTg‐AD mice were subjected to left‐lateral closed‐head impact injury at 10‐12 weeks of age (Tagge CA, et al.,2018). At six months post‐TBI, mice were sacrificed using transcardial perfusion. The harvested brains were submerged in 10% formalin for 24 hours and stored in Gadavist‐doped PBS (1:400 dilution) until MRI. MRI data were acquired using a 9.4T Bruker scanner and a 2‐element cryoprobe. Key parameters are TR=300ms, TE=27.7ms, b=3000 and 5000 s/mm^2^ (64 directions), FOV=13.92x10.20x7.20mm^3^, Matrix=232x170x120, resolution=60mm^3^. Diffusion MRI was analyzed in DSI Studio (http://dsistudio.labsolver.org). Age‐ and gender‐matched 3xTg‐AD mice without TBI were used as controls.

**Result:**

Figure 1 shows the representative quantitative anisotropy (QA) maps obtained from a mouse with TBI. At 6 months post‐TBI, decreased QA values were observed in the brain regions ipsilateral to the injury (arrows) compared to the corresponding contralateral regions. Figure 2 shows the representative QA maps of the regions around the hippocampus in mice with and without TBI. Compared to the control mouse, the mouse with neurotrauma showed decreased QA values in the corpus callosum and bilateral hippocampus, especially in the CA1 subregion.

**Conclusion:**

Our results demonstrated that ultra‐high resolution diffusion MRI can capture subtle alterations of white matter integrity in different brain sub‐regions. Using this technique, we were able to detect subtle white matter degeneration in a mouse model of Alzheimer’s disease. The observed white matter deterioration may contribute to the worsening of Alzheimer’s disease after neurotrauma.